# Diuretic Resistance Prediction and Risk Factor Analysis of Patients with Heart Failure During Hospitalization

**DOI:** 10.5334/gh.1113

**Published:** 2022-05-27

**Authors:** Xiao Lu, Yi Xin, Jiang Zhu, Wei Dong, Tong-Peng Guan, Jia-Yue Li, Qin Li

**Affiliations:** 1 Department of Biomedical Engineering, School of Life Science, Beijing Institute of Technology, Beijing 100081, China; 2 Department of Cardiology, the Sixth Medical Centre, Chinese PLA General Hospital, Beijing 100081, China

**Keywords:** diuretic resistance, machine learning, prediction, risk factors, decompensated heart failure

## Abstract

**Objectives:**

This study performed a prediction and risk factor analysis of diuretic resistance (DR) in patients with decompensated heart failure during hospitalization.

**Methods:**

The data of patients with decompensated heart failure treated in 2010–2018 with DR (n = 3,383) or without DR (n = 15,444) were retrospectively collected from Chinese PLA General Hospital medical records. Statistical analysis of baseline was performed on two groups of people, and the risk factor of DR was analyzed through logic regression. Six machine learning models were built accordingly, and the adjustment of model super parameters was performed by using Bayesian optimization method. Finally, the optimal algorithm was selected according to prediction efficiency.

**Results:**

The preliminary analysis of variance showed significant differences in the incidence of DR among patients with lung infection, hyperlipidemia, type 2 diabetes, and kidney disease. There were significant differences in estimated glomerular filtration rate (eGFR) (P < 0.001). In addition, some physical indicators like BMI were different, the laboratory results like mean red blood cell volume or C-reactive protein assay were also significantly different. The optimal classification model indicated that the best cutoff points for risk factors were vein carbon dioxide, 21 mmol/L and 29 mmol/L; total protein, 64 g/L; pro-brain natriuretic peptide (pro-BNP), 7,600 pg/mL; eGFR, 50 mL/(min ∙ 1.73 m^2^); serum albumin, 33 g/L; hematocrit, 0.32% and 0.56%; red blood cell volume distribution width, 13; and age, 59 years. The optimal area under the curve was 0.9512. The ranked features derived from the model were age, abnormal sodium level, pro-BNP level, serum albumin level, D-dimer level, direct bilirubin level, and eGFR.

**Conclusions:**

The DR risk prediction model based on a gradient boosting decision tree created here identified its important risk factors. The model made very accurate predictions using simple indicators and simultaneously calculated cutoff values to help doctors predict the occurrence of DR.

## Introduction

Heart failure, a complex syndrome that develops in the terminal stage of cardiovascular disease, seriously threatens patient life and health. More than 26 million people worldwide are hospitalized annually for acute heart failure [1,2,3], with a one-year mortality rate of 20%–30% and increased risk of rehospitalization that create huge public health and financial burdens [4,5].

Congestion, the most frequent (70%) clinical manifestation is characterized by volume overload and insufficient cardiac output [6,7]. Its related symptoms include exertional shortness of breath, orthopnea, paroxysmal nocturnal dyspnea, fatigue, tissue congestion (such as peripheral edema), and increased pigmentation. Diuretics (especially loop diuretics) are pivotal in relieving congestion and its related symptoms in patients with heart failure [8].Diuretics are the recommended first-line therapy for acute heart failure [9,10,11,12], the efficacy of which is determined based on the patient’s response to its administration. The optimal diuretic response can be measured by urinary output, which is defined as a negative fluid balance of at least 1.5–2 L over 24 h after administration. Therefore, diuretic resistance (DR) may be defined as an inadequate response and ineffective decongestion despite maximum-dose diuretic therapy [13,14].

Understanding the cause of DR is pivotal to its reversal. There can be many reasons for DR; the efficacy of a diuretic depend on its delivery to its intraluminal site of action (pharmacokinetics) as well as the dynamics of its interaction with its receptor at the site of action (pharmacodynamics)[15]. Aronson provided a detailed introduction to the use of diuretics and the relationship between DR and renal ion transport that proves DR complexity [16]. Diuretic resistance implies a failure to increase fluid and sodium (Na^+^) output sufficiently to relieve volume overload, edema, or congestion, despite escalating doses of a loop diuretic to a ceiling level. It is a major cause of recurrent hospitalizations in patients with chronic heart failure and predicts death but is difficult to diagnose unequivocally [10]. Despite the fact that diuretics themselves are not linked to increased survival, diuretic efficacy has been shown to prolong event-free survival, regardless of glomerular filtration rate [17].

Because DR contributes to worsening heart failure and poor outcomes, great effort is directed toward identifying the best therapeutic strategies [18]; at the same time, it is equally important to predict the occurrence of DR and identify its risk factors. This information could aid the early treatment of patients with DR and improve their prognosis.

## Methods

### Data collection and study design

**Figure 1:**
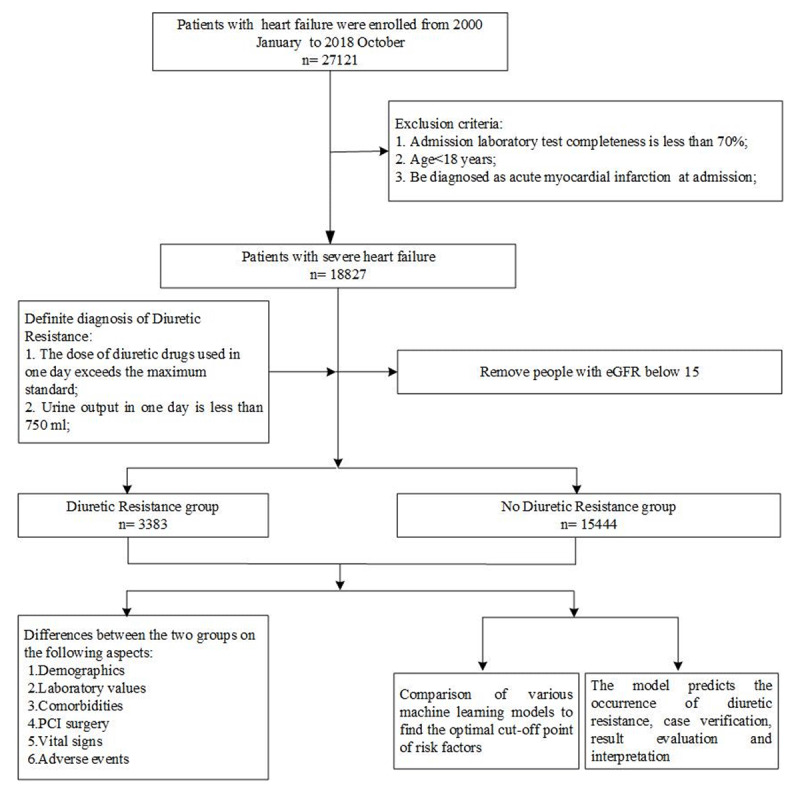
**Flow chart of the study population enrollment.** eGFR: estimate glomerular filtration rate.

Data obtained from the electronic medical records from the People’s Liberation Army General Hospital, 2010–2018, included patients’ demographic and clinical characteristics, surgical history, nursing records, and laboratory examination data. These patients were hospitalized due to cardiac decompensation and were treated in the Department of Cardiology. Data were collected from bedside instrumentation monitoring and routine in-hospital tests. These data were gathered together by computer programming methods to form the dataset we analyzed. The patients’ baseline characteristics at hospital admission were included in the analysis. The outcome was the occurrence of DR during hospitalization. Patients for whom demographic data were incomplete, with less than 70% completeness of the selected observation indicators, an estimated glomerular filtration rate (eGFR) <15 (min ∙ 1.73 m^2^), who were diagnosed with acute myocardial infarction were excluded (***Figure 1***).

Ultimately, the preliminary statistical analysis included 3,383 diuretic-resistant samples and 15,444 non-diuretic-resistant samples. Logic regression and six machine learning algorithm models were implemented to analyze the risk factors and obtain the best cutoff point for the risk factors.

### Definitions and baseline characteristics

DR during hospitalization was defined as insufficient decongestion despite the administration of the following: (1) oral or intravenous furosemide >160 mg or >80 mg in one day; and/or (2) oral or intravenous torsemide >80 mg or >40 mg in one day; and/or (3) intravenous bumetanide >4 mg in one day [19,20]. Simultaneously, the total urine output of ≤750 mL within 24 h after diuretic administration. Potassium abnormalities and sodium abnormalities were mainly considered electrolyte disorders. During the hospitalization, abnormal potassium level was defined as a serum potassium level exceeding the normal range of 3.5–5.5 mmol/L for more than three measurements, while serum sodium abnormalities was defined as a serum sodium level exceeding the normal range of 135–145 mmol/L for more than three measurements. The occurrence of DR was determined after hospitalization, and the ion disturbance index was determined prior to the time of diagnosis of DR as a predictor.

We included patients with heart failure coded as International Classification of Diseases, 9^th^ edition codes 402, 404, and 428. Patients with decompensated heart failure who were aged >18 years at the time of admission and had a New York Heart Association heart function classification of level 3 or above were included in the analysis. Heart failure and related knowledge were defined based on American College of Cardiology/American Heart Association and the European Society of Cardiology guidelines [20,21,22,23].

The patients’ baseline characteristics were the first valid recorded values after hospital admission. All the indicators used for prediction were the baseline indicators of the patient at the time of admission, and the ion fluctuations were also the data measured before the diagnosis of DR. Hypertension, hyperlipidemia, kidney disease, pulmonary infection, and tumor were obtained from the history diagnosis results in the electronic medical record system. Body mass index (BMI) was calculated as follows: BMI = weight (kg)/height (m^2^). The eGFR was calculated through MRDR calculation formula as previously described: eGFR [mL/(min × 1.73 m^2^)] = 186 × serum creatinine (mg/dL) – 1.154 × age – 0.203(×0.742 if female)[24].

### Statistical analysis

The demographic and baseline characteristics of the study population were compared using the Pearson chi-square test for categorical variables and Student’s *t*-test for continuous variables. Normality tests were performed using the Shapiro–Wilk test. Normally distributed continuous variables, non-normally distributed continuous variables, and categorical variables are expressed as mean ± standard deviation, quartiles, and count or percentage, respectively; differences were detected using the two-sample independent *t*-test, rank sum test, and chi-square test, respectively. The difference between eGFR and categorical variables in the occurrence of DR was analyzed using binary logistic regression, and the odds ratio (OR) and 95% confidence interval (CI) was calculated. SPSS software for Windows (version 25.0, SPSS Inc., Chicago, IL, USA) was used for the statistical analyses.

### Classification prediction model

We randomly split the data of DR and non-DR patients by a 7:2:1 ratio for training, internal validation, and testing, respectively, and 10-fold cross validation was adopted. Different machine-learning classification algorithms were used for the classification analysis. The six algorithms were gradient boosting decision tree (GBDT), extreme gradient boosting, random forest, light gradient boosted machine, decision tree, and support vector machine. For model evaluation, we added five evaluation indexes including precision, recall, F1-score, accuracy and AUC. As for parametric tuning, we used Bayesian optimization method to automatically adjust the model hyperparameters. The predictive capabilities of the different machine learning algorithms through the 5 evaluation index enabled the more reliable calculation of the precise cutoff values of the indicators. Shapley additive explanation (SHAP) value analysis graphs were used to show the specific values of the turning points of the important biological detection indicators to provide better guidance and advice for clinical judgment [25]. The SHAP value of each indicator indicates the impact on the model output and was calculated according to the GBDT model. Each blue point in the SHAP value analysis graph represents a sample. A SHAP value above 0 indicates the probability of DR. The SHAP installation package and the machine learning model packages were imported in a python 3.7 environment, and can be learned from the official website: *https://shap.readthedocs.io/en/latest/api.html*.

## Results

### Differences in baseline characteristics between DR and non-DR patients with heart failure

The baseline characteristics of the total population and the groups stratified by the occurrence of DR are presented in ***Table 1***. Of the population, 17.97% developed DR. Women had a higher DR rate than men. More DR patients had hypokalemia, hyponatremia, pulmonary infection, type 2 diabetes mellitus, and kidney disease, whereas a smaller proportion suffered from hyperlipidemia, and there were no significant differences in the incidence of tumor and hypertension. Patients with DR had a lower blood pressure and BMI. Pro-brain natriuretic peptide (BNP), troponin T, and international normalized (IN) ratio levels were very high in patients with DR, indicating that these patients had advanced heart failure. Concurrently, the mean direct bilirubin level, which reflects congested liver function, was higher in the DR group. Elevated creatinine and reduced eGFR also indicated poor kidney function in patients with DR. According to the four CFR staging derived from eGFR values (eGFR below 15 was excluded), the proportion of patients with diuretic resistance was significantly lower in stageI–II and significantly higher in stage III–IV. Fewer DR patients had undergone percutaneous coronary intervention. The all-cause mortality rate was 20.1%, while that of the non-DR group was only 3.7%.

**Table 1:** Patients’ baseline clinical characteristics by study group.


CHARACTERISTICS	WITHOUT DR GROUP (n = 15,444)	WITH DR GROUP (n = 3,383)	*p* VALUE

**Demographic**			

Gender(Male),n(%)	9,389(60.8%)	1,920(56.8%)	<0.001

Age	66.84 ± 15.40	67.01 ± 16.86	0.013

**Comorbidities,n(%)**			

Hypokalemia	2,351(15.2%)	657(19.4%)	<0.001

Hyponatremia	1,839(11.9%)	850(25.1%)	<0.001

Tumor	845(5.5%)	187(5.5%)	0.896

Pulmonary infection	1,486(9.6%)	631(18.7%)	<0.001

Hyperlipidemia disease	1,135(7.3%)	154(4.6%)	<0.001

Hypertension	3,664(23.7%)	762(22.5%)	0.136

T2DM	4,405(28.5%)	1,121(33.1%)	<0.001

kidney disease	1,140(7.4%)	575(17.0%)	<0.001

**Physical**			

BMI	24.34 ± 4.07	23.98 ± 4.23	<0.001

DBP(mmHg)	74.06 ± 12.76	73.27 ± 13.78	<0.001

SBP(mmHg)	129.49 ± 20.30	127.69 ± 23.05	<0.001

**Laboratory Results**			

C-reactive protein assay(mg/dl)	1.91(0.34–2.72)*	2.51(0.53–4.0)*	<0.001

Glutamyl transferase(U/L)	35.6(21.4–62.05)*	47.7(25.1–87.2)*	<0.001

Neutrophil (%)	0.67 ± 0.12	0.74 ± 0.14	<0.001

Lactate dehydrogenase(U/L)	189.5(158.2–247.5)*	242.5(188.1–327.9)*	<0.001

Carbon dioxide (mmol/L)	25.89 ± 3.78	24.86 ± 4.98	<0.001

LDL-C(mmol/L)	2.34 ± 0.79	2.27 ± 0.92	<0.001

IN Ratio	1.21 ± 0.51	1.35 ± 0.75	<0.001

Aspartate aminotransferase(U/L)	20.3(15.7–28.9)*	23.3(16.5–38.3)*	<0.001

Mean RBC volume (fL)	91.46 ± 6.17	91.43 ± 6.91	0.72

RBC hemoglobin concen.(g/L)	332.26 ± 14.42	329.83 ± 16.25	<0.001

TC(mmol/L)	3.98 ± 1.02	3.88 ± 1.21	<0.001

Total protein(g/L)	66.63 ± 7.47	64.02 ± 9.33	<0.001

Inorganic phosphorus (mmol/L)	1.16(0.98–1.27)*	1.17(0.96–1.35)*	<0.001

Chloride (mmol/L)	102.97 ± 5.14	101.47 ± 6.48	<0.001

Lymphocyte (%)	0.24 ± 0.10	0.18 ± 0.11	<0.001

WBC (10e9/L)	6.6(5.27–8.2)*	7.46(5.54–10.23)*	<0.001

Direct bilirubin (umol/L)	4.4(2.9–7.2)*	5.3(2.9–10.5)*	<0.001

AP (U/L)	70.3(56.6–84.4)*	77.4(60.2–100.6)*	<0.001

RDW (%)	14.14 ± 1.89	15.02 ± 2.31	<0.001

HCT (%)	0.38 ± 0.07	0.35 ± 0.08	<0.001

RBC (10e12/L)	4.18 ± 0.77	3.81 ± 0.89	<0.001

Creatinine (umol/L)	92.31 ± 41.26	117.43 ± 59.14	<0.001

Troponin T (ng/mL)	0.04(0.02–0.31)*	0.05(0.02–0.20)*	<0.001

pro-BNP (pg/mL)	3238(867.2–6726.3)*	6470(2230–10513)*	<0.001

Glucose (mmol/L)	6.60 ± 2.92	7.62 ± 3.88	<0.001

PLT (10e9/L)	193.47 ± 76.81	184.17 ± 88.51	<0.001

D-dimer (μg/mL)	1.88 ± 2.97	3.27 ± 4.21	<0.001

PT (s)	15.01 ± 4.03	16.27 ± 5.75	<0.001

PTA (%)	77.40 ± 16.12	71.76 ± 18.51	<0.001

FIB (mg/dL)	3.59(2.92–4.65)*	3.88(2.99–5.09)*	<0.001

Serum albumin (g/L)	37.57 ± 4.98	34.38 ± 5.69	<0.001

HGB (g/L)	126.90 ± 23.74	114.58 ± 27.19	<0.001

Serum calcium (mmol/L)	2.22 ± 0.16	2.15 ± 0.20	<0.001

Serum natrium (mmol/L)	139.86 ± 4.61	138.09 ± 5.76	<0.001

MCH (pg)	30.39 ± 2.41	30.17 ± 2.70	<0.001

Thrombin time (s)	17.11 ± 4.38	17.45 ± 4.72	0.01

Amylase (U/L)	72.4(47.2–73.6)*	54.5(37.4–73.6)*	<0.001

Creatine kinase isoenzymes (ng/mL)	3.77(1.86–9.23)*	3.39(1.96–9.23)*	0.011

eGFR (min * 1.73 m^2^)	68.37 ± 31.37	57.37 ± 33.95	<0.001

CRF Staging			

I	2925(18.9%)	474(14.0%)	0.010

II	5933(38.4%)	837(24.7%)	<0.001

III	5469(35.4%)	1327(39.2%)	0.013

IV	1117(7.2%)	745(22.0%)	<0.001

**PCI surgery (YES), n(%)**	2119(13.7%)	303(9.0%)	<0.001

**Death in-hospital (YES), n(%)**	523(3.4%)	728(21.5%)	<0.001


* Presented as median (interquartile range). Categorical variables are presented as n (%). Continuous variables are presented as mean ± standard deviation. AP, alkaline phosphatase; BMI, body mass index; DBP, diastolic blood pressure; D-dimer, plasma D-dimer assay; eGFR, estimated glomerular filtration rate; FBG, fasting blood glucose; FIB, plasma fibrinogen; HCT, hematocrit; HGB, hemoglobin determination; IN ratio, international normalized ratio; LDL-C, low-density lipoprotein cholesterol; MCH, mean corpuscular hemoglobin;CRF, chronic renal failure; PCI, percutaneous coronary intervention; PLT, platelet count; pro-BNP, pro-brain natriuretic peptide; PT, plasma prothrombin time determination; PTA, plasma prothrombin activity; RBC, red blood cell count; RDW, red blood cell volume distribution width; SBP, systolic blood pressure; TC, total cholesterol; T2MD, type 2 diabetes mellitus; WBC, white blood cell count.

### Analysis of risk factors for DR by logistic regression

**Figure 2:**
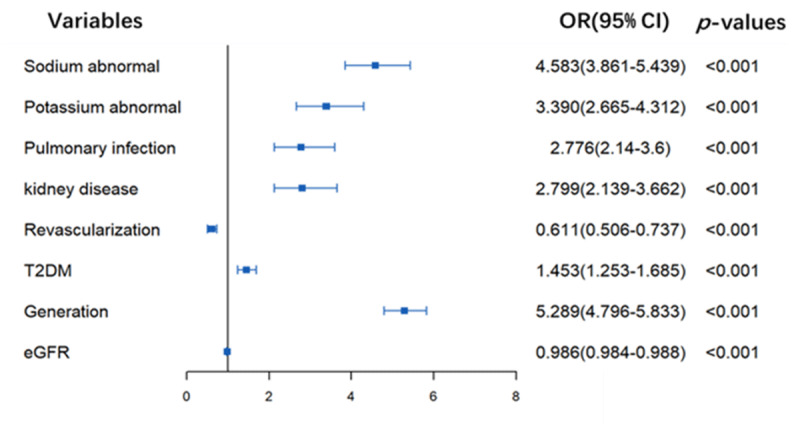
**Logistic regression analysis of risk factors for diuretic resistance.** OR: odds ratio; CI: confidence interval; Generation: the age division. For continuous variables: eGFR, the ORs means the risk per unit increased.

Binary logistic regression modeling was performed for the risk factor analysis. As shown in ***Figure 2***, abnormal sodium and potassium levels increased the risk of DR (OR, 4.583; 95% CI, 3.861–5.439; P < 0.001; and OR, 3.390; 95% CI, 2.665–4.312; P < 0.001, respectively). Pulmonary infection and kidney disease also increased the risk of DR (OR, 2.776; 95% CI, 2.14–3.6; P < 0.001; and OR: 2.799 (2.139–3.662); P < 0.001, respectively). The population was divided into four age groups: ≤45, 45–65, 65–75, and ≥75 years, showed as generation; each additional unit increased the OR to 5.289. eGFR was negatively proportional to DR, the smaller the eGFR value, the more likely DR occurred.

### Best cutoff point for risk factors identified by the optimal classification model

**Figure 3:**
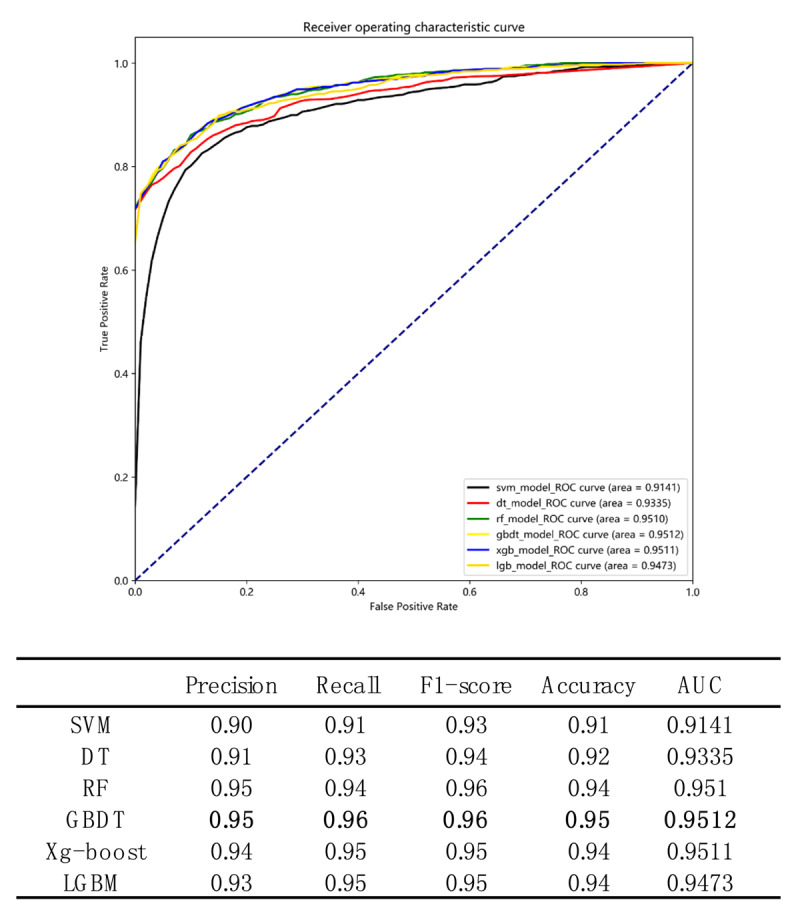
**Comparison of prediction performance of six machine learning classification algorithms.** The six algorithms include Gradient Boosting Decision Tree (GBDT), Extreme Gradient Boosting (Xg-boost), Random Forest, Light-GBM, Decision Tree, and Support Vector Machine (SVM). The ROC curve of GBDT is shown by the yellow line, the ROC curve of Xg-boost is shown by the blue line, the ROC curve of Random Forest is shown by the green line, the ROC curve of Light-GBM is shown by the orange line, the ROC curve of Decision Tree is shown by the red line, the ROC curve of SVM is shown by the black line.

**Figure 4:**
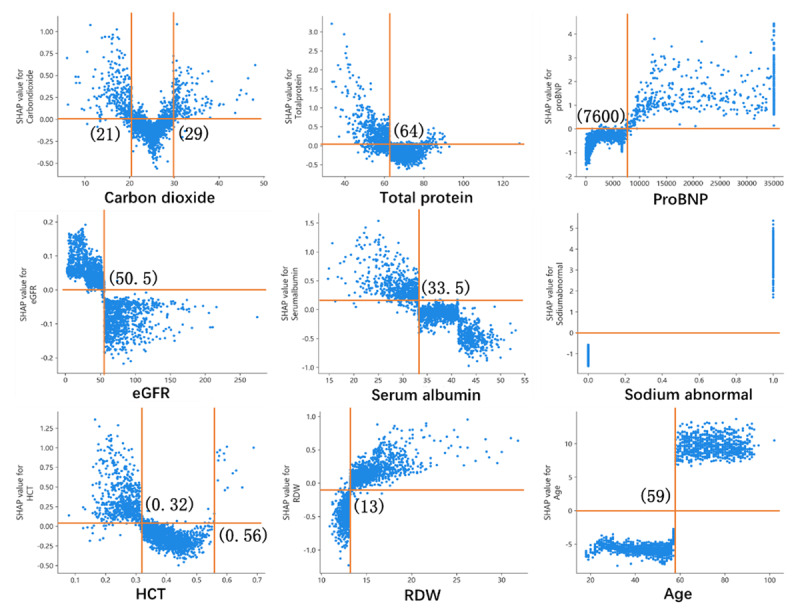
**The optimal classification model obtains the best cutoff point of risk factors for diuretic resistance.** eGFR: estimate glomerular filtration rate; HCT: hematocrit; RDW: blood cell volume distribution width; pro-BNP: pro-brain nitric peptide.

As shown in ***Figure 3***, six machine learning algorithm models were implemented to classify the patients into DR and non-DR groups. According to the values of comprehensive indexes F1-score and AUC, GBDT is the model with the best performance (F1-score is 0.96, and the AUC is 0.9512). According to the optimal model, the best cutoff point for the risk factors was calculated (***Figure 4***). The optimal classification model indicated that the best cutoff points for risk factors were: vein carbon dioxide, 21 and 29 mmol/L; total protein, 64 g/L; pro-BNP, 7,600 pg/mL; eGFR, 50 mL/(min × 1.73 m^2^); serum albumin, 33 g/L; hematocrit (HCT), 0.32% and 0.56%; blood cell volume distribution width, 13; and age, 59 years (***Figure 4***).

### Introduction to DR prediction model and interpretability

**Figure 5:**
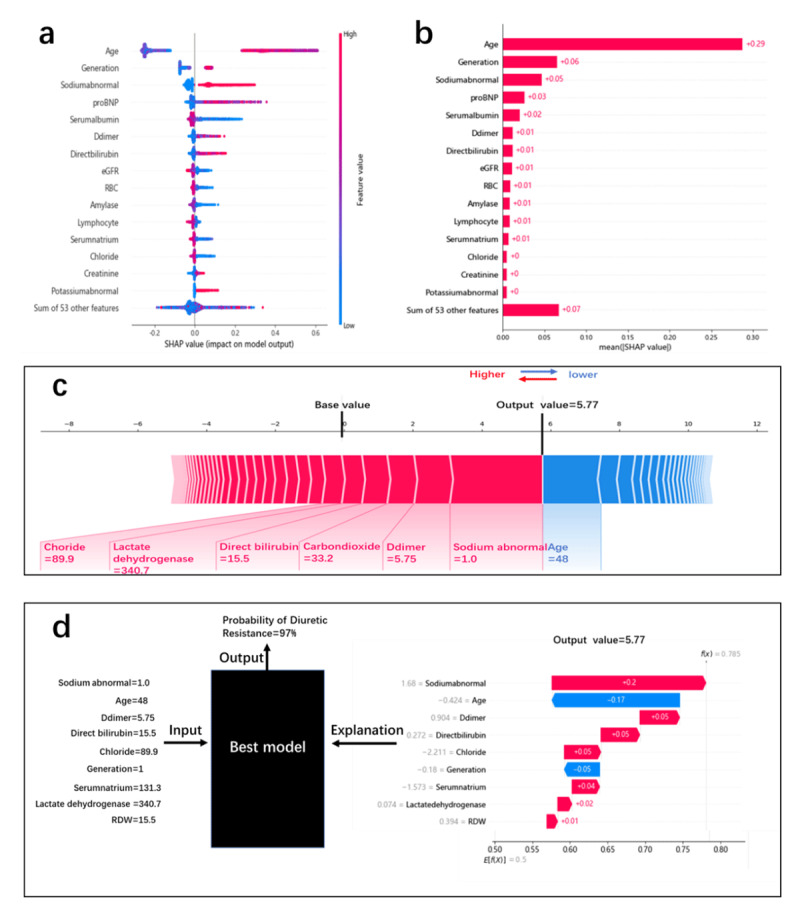
(**a**): SHAP summary graph and the distribution of SHAP values for each sample. (**b**): SHAP value contribution graph of each indicator of a single sample. (**c**): Characteristic SHAP value influence diagram of a single sample. (**d**): Model prediction diagram and explanation for individual.

We calculated the importance of each feature according to the SHAP values (***Figure 5A,B***). The SHAP value is used to calculate the marginal contribution of a feature when it is added to the model, and the different marginal contributions of the feature in the case of all feature sequences [26]. Each point shown in ***Figure 5A*** represents the SHAP value of one feature of an instance. The position on the y-axis was determined by the feature, while the position on the x-axis was determined by the SHAP value. The color represents the feature’s value from small to large, which allows understanding of the distribution of the SHAP values for each feature. ***Figure 5B*** presents the feature importance ranking, showing that age; sodium abnormality; pro-BNP, serum albumin, D-dimer, and direct bilirubin levels; and eGFR are more important. ***Figure 5C*** shows that we used the SHAP to explain the prediction of a single sample. Each feature value is a force that increases or decreases the DR likelihood. The prediction starts from the baseline, which is the average of all predictions, and each SHAP value is an arrow that increases (positive value) or decreases (negative value) the prediction. ***Figure 5D*** shows the probability of DR after a patient’s indicators are entered in the model and the impact of each indicator on the model’s results. The random input of a patient’s indicators shows that the probability of this patient suffering from DR is 97%. We verified that DR indeed occurred, indicating the model’s reliability.

## Discussion

The current study revealed the differences between DR and non-DR patients in comorbidities and most test indicators. This study included more detailed observation characteristics than previous studies.

The main finding of this study is that the application of machine learning to predict DR prediction is more efficient than traditional statistical analysis and can find the best cutoff value that will provide doctors with more advisable information. Interestingly, the optimal model accurately predicted the probability of a person developing DR and provided the relevant indicators’ importance. A sub-analysis of the Organized Program to Initiate Lifesaving Treatment in Hospitalized Patients with Heart Failure (OPTIMIZE-HF) illustrated that 20% of patients had hyponatremia at the time of admission [27], a finding that is consistent with our finding. Hyponatremia in hospitalized patients with heart failure is associated with longer hospital stays and higher in-hospital and early post-discharge mortality rates, which may reflect worsening heart failure and the deleterious effects of neurohormone activation [28]. We also found that a higher proportion of patients with DR had hypokalemia at baseline. A review reported that mortality risk progressively increased with dyskalemia and was differentially greater in patients with HF, taking diuretics, and taking renin-angiotensin-aldosterone system inhibitors related to hypokalemia and hyperkalemia, respectively [29]. Many studies have shown a relationship between heart failure and diabetes; the risk of heart failure in patients with diabetes is more than twice that in non-diabetic patients [30,31]. In our univariate analysis, a higher proportion of patients with DR heart failure had diabetes. The pathophysiology of the underlying individual differences in the diuretic response and the specific mechanism of DR vary. Research has shown that renal tubular resistance is the primary driver of loop DR in acute heart failure and that kidney disease and renal dysfunction can increase the DR risk [32]. To rule out the bias of severe kidney disease on the analysis of the results, we eliminated the population with an eGFR < 15 (min ∙ 1.73 m^2^).

Predicting whether a patient will develop DR at an early stage of admission is important since it facilitates the adjustment of drugs, relieves dyspnea sooner, shortens the length of hospital stay, and reduces the risk of readmission. Valente et al. previously reported the association of a poor diuretic response with more advanced heart failure, renal impairment, diabetes, and atherosclerotic disease [33]. Many clinical variables and biomarkers are also related to diuretic response. Our analysis showed that biomarkers such as pro-BNP, troponin T, and IN ratio, which were very high in patients with DR, reflected the serious degree of heart failure in this population. Concurrently, the mean direct bilirubin level, which reflects liver congestion, was also higher in the DR group. Elevated creatinine level and a reduced eGFR also indicated poor kidney function in people with DR. Interestingly, we found two cutoff values for vein carbon dioxide: <21 mmol/L or >29 mmol/L increased the risk of DR. These biological indicators are different from previous findings because we considered more common and easily available indicators to train the classification models, which are different from previous cognitions, and used machine learning classification models to produce novel results. We traditionally considered 65 years of age as a demarcation point, but for patients with decompensated heart failure, DR is likely to occur after 59 years of age, which was also the strongest predictor. Another study found that creatinine, neutrophil gelatinase-associated lipocalin, endothelial cell-selective adhesion molecule, and lymphotoxin beta receptor are strongly associated with DR [34].

In our research, SHAP was used to explain the model to obtain the influence index of this result. By inputting the observed indicators of one person into the model, we were able to quickly predict the probability of DR at the initial admission. This could help in the early treatment of patients with DR, which might improve their prognosis.

In conclusion, here we constructed a DR risk prediction model based on a GBDT that can identify important risk factors. The model can make very accurate predictions for individuals based on simple indicators. Simultaneously, the model can calculate cutoff values to help doctors judge the occurrence of DR.

## Limitations

Since this is a retrospective data analysis, the drug used by patients with heart failure is very complicated, and our research has not been included in the drug analysis, which is a limitation. Additionally, this was a single-center observational study and not a randomized controlled trial; as such, although the total dataset was large, unforeseen confounding factors could have affected the results. We currently only predicted the occurrence of DR in hospitals; in the future, we will continue to study and analyze the applicability of diuretic drugs for avoiding adverse events.

## Data Accessibility Statement

Since the data used in this study is the medical data of the military hospital, involving the management of the army, so our data cannot be copied and disclosed.

## Abbreviations

BMI, body mass index; D-dimer, plasma D-dimer assay; DR, diuretic resistance; eGFR, estimated glomerular filtration rate; IN ratio, international normalized ratio; pro-BNP, pro-brain natriuretic peptide; SHAP, SHapley Additive explanation; Generation, the age division.

## References

[1] Benjamin EJ, Blaha MJ, Chiuve SE, et al. Heart disease and stroke statistics—2017 update: A report from the American Heart Association. Circulation. 2017; 135: e146–e603. DOI: 10.1161/CIR.0000000000000491 28122885 PMC5408160

[2] Maggioni AP. Epidemiology of heart failure in Europe. Heart Fail Clin. 2015; 11: 625–35. DOI: 10.1016/j.hfc.2015.07.015 26462102

[3] Kurmani S, Squire I. Acute heart failure: definition, classification and epidemiology. Current Heart Failure Reports. 2017; 14: 385–92. DOI: 10.1007/s11897-017-0351-y 28785969 PMC5597697

[4] Chen J, Hsieh AF, Dharmarajan K, et al. National trends in heart failure hospitalization after acute myocardial infarction for Medicare beneficiaries: 1998–2010. Circulation. 2013; 128: 2577–84. DOI: 10.1161/CIRCULATIONAHA.113.003668 24190958 PMC4415510

[5] Shen L, Jhund PS, Anand IS, et al. Developing and validating models to predict sudden death and pump failure death in patients with heart failure and preserved ejection fraction. Clin Res Cardiol. 2021; 110: 1234–48. DOI: 10.1007/s00392-020-01786-8 33301080 PMC8318942

[6] Mullens W, Damman K, Harjola VP, et al. The use of diuretics in heart failure with congestion—a position statement from the Heart Failure Association of the European Society of Cardiology. European Journal of Heart Failure. 2019; 21: 137–55. DOI: 10.1002/ejhf.1369 30600580

[7] Costanzo MR. The cardiorenal syndrome in heart failure. Heart Failure Clinics. 2020; 16: 81–97. DOI: 10.1016/j.hfc.2019.08.010 31735318

[8] Faris RF, Flather M, Purcell H, et al. Diuretics for heart failure. Cochrane Database Syst Rev. 2012: Cd003838. DOI: 10.1002/14651858.CD003838.pub3 22336795

[9] Setoguchi S, Stevenson LW, Schneeweiss S. Repeated hospitalizations predict mortality in the community population with heart failure. Am Heart J. 2007; 154: 260–6. DOI: 10.1016/j.ahj.2007.01.041 17643574

[10] Wilcox CS, Testani JM, Pitt B. Pathophysiology of Diuretic Resistance and Its Implications for the Management of Chronic Heart Failure. Hypertension. 2020; 76: 1045–54. DOI: 10.1161/HYPERTENSIONAHA.120.15205 32829662 PMC10683075

[11] McDonagh TA, Metra M, Adamo M, et al. 2021 ESC Guidelines for the diagnosis and treatment of acute and chronic heart failure. Eur Heart J; 2021. 10.1093/eurheartj/ehab853 34922348

[12] Savarese G, Stolfo D, Sinagra G, et al. Heart failure with mid-range or mildly reduced ejection fraction. Nat Rev Cardiol. 2022; 19: 100–16. DOI: 10.1038/s41569-021-00605-5 34489589 PMC8420965

[13] Suri SS, Pamboukian SV. Optimal diuretic strategies in heart failure. Annals of Translational Medicine. 2021; 9: 517. DOI: 10.21037/atm-20-4600 33850914 PMC8039650

[14] Cox ZL, Testani JM. Loop diuretic resistance complicating acute heart failure. Heart Failure Reviews. 2020; 25: 133–45. DOI: 10.1007/s10741-019-09851-9 31520280

[15] Brater DC. (ed.). Update in diuretic therapy: clinical pharmacology. Seminars in nephrology; 2011: Elsevier. DOI: 10.1016/j.semnephrol.2011.09.003 22099505

[16] Aronson D. The complexity of diuretic resistance. Wiley Online Library; 2017. DOI: 10.1002/ejhf.815 28370822

[17] Jardim SI, Ramos Dos Santos L, Araujo I, et al. A 2018 overview of diuretic resistance in heart failure. Rev Port Cardiol (Engl Ed). 2018; 37: 935–45. DOI: 10.1016/j.repc.2018.03.014 30470451

[18] Valente M, Voors A, Damman K, et al. Diuretic resistance in acute heart failure-clinical characteristics and prognostic significance. European Heart Journal. 2013; 34. DOI: 10.1093/eurheartj/eht308.1729 24585267

[19] ter Maaten JM, Valente MA, Damman K, et al. Diuretic response in acute heart failure-pathophysiology, evaluation, and therapy. Nat Rev Cardiol. 2015; 12: 184–92. DOI: 10.1038/nrcardio.2014.215 25560378

[20] Rahman R, Paz P, Elmassry M, et al. Diuretic Resistance in Heart Failure. Cardiol Rev. 2021; 29: 73–81. DOI: 10.1097/CRD.0000000000000310 32282394

[21] Clarke B. Cardiorenal Interactions, Diuretic Resistance, and Acute Heart Failure: Renal Response vs Renal Function. Can J Cardiol. 2019; 35: 1079–81. DOI: 10.1016/j.cjca.2019.02.020 31472805

[22] Cox ZL, Hung R, Lenihan DJ, et al. Diuretic Strategies for Loop Diuretic Resistance in Acute Heart Failure: The 3T Trial. JACC Heart Fail. 2020; 8: 157–68. DOI: 10.1016/j.jchf.2019.09.012 31838029 PMC7058489

[23] Kumar A, Singh V. Atherogenic dyslipidemia and diabetes mellitus: What’s new in the management arena? Vascular health and risk management. 2010; 6: 665. DOI: 10.2147/VHRM.S5686 20859538 PMC2941780

[24] Correction: Using Standardized Serum Creatinine Values in the Modification of Diet in Renal Disease Study Equation. Ann Intern Med. 2021; 174: 584. DOI: 10.7326/L21-0010 33872525

[25] Lundberg SM, Lee S-I. (eds.) A unified approach to interpreting model predictions. Proceedings of the 31st international conference on neural information processing systems; 2017.

[26] Lundberg SM, Erion GG, Lee S-I. Consistent individualized feature attribution for tree ensembles. arXiv preprint arXiv:180203888. 2018.

[27] Gheorghiade M, Abraham WT, Albert NM, et al. Relationship between admission serum sodium concentration and clinical outcomes in patients hospitalized for heart failure: An analysis from the OPTIMIZE-HF registry. European Heart Journal. 2007; 28: 980–8. DOI: 10.1093/eurheartj/ehl542 17309900

[28] Chatterjee K. Hyponatremia in heart failure. J Intensive Care Med. 2009; 24: 347–51. DOI: 10.1177/0885066609344941 19850560

[29] Collins AJ, Pitt B, Reaven N, et al. Association of Serum Potassium with All-Cause Mortality in Patients with and without Heart Failure, Chronic Kidney Disease, and/or Diabetes. Am J Nephrol. 2017; 46: 213–21. DOI: 10.1159/000479802 28866674 PMC5637309

[30] Berg DD, Wiviott SD, Scirica BM, et al. A Biomarker-Based Score for Risk of Hospitalization for Heart Failure in Patients With Diabetes. Diabetes Care; 2021. DOI: 10.2337/figshare.16451109 PMC8546278 34535469

[31] Ter Maaten JM, Rao VS, Hanberg JS, et al. Renal tubular resistance is the primary driver for loop diuretic resistance in acute heart failure. Eur J Heart Fail. 2017; 19: 1014–22. DOI: 10.1002/ejhf.757 28105769 PMC6231236

[32] Ter Maaten JM, Rao VS, Hanberg JS, et al. Renal tubular resistance is the primary driver for loop diuretic resistance in acute heart failure. European Journal of Heart Failure. 2017; 19: 1014–22. DOI: 10.1002/ejhf.757 28105769 PMC6231236

[33] Valente MA, Voors AA, Damman K, et al. Diuretic response in acute heart failure: clinical characteristics and prognostic significance. European Heart Journal. 2014; 35: 1284–93. DOI: 10.1093/eurheartj/ehu065 24585267

[34] Ter Maaten JM, Valente MA, Metra M, et al. A combined clinical and biomarker approach to predict diuretic response in acute heart failure. Clinical Research in Cardiology. 2016; 105: 145–53. DOI: 10.1007/s00392-015-0896-2 26280875 PMC4735256

